# Obstetrics

**Published:** 1876-04

**Authors:** 


### VI. Obstetrics.

1. *Siamese Obstetrics.* Dr. W. L. Hutchinson, of Bangkok, Siam. (*N. Y. Med. Rec.*, Feb., 1876.)

The old "*yi*" or midwife superintends the manipulations and pressures made by friends of the patient, upon the occasion of her lying in. The woman is placed upon her back, and then a female friend is seated on each side ; the latter press forcibly upon the uterus down and backward. If three or five hours of this procedure fail to expel the fœtus, then one of the attendants, steadied by her colleague, tramps upon the abdomen of the laboring woman, always treading anatomically above the uterus.

This failing, the last resort is to suspend the unfortunate by a strap beneath the arms. She is then clasped by the arms of one or both of the attendants, and the baby is *squeezed out* of the womb, or else something gives way inside the poor laboring woman, uterus, perineum or the baby's cranium. Comment on some of the horrible results of this high art is superfluous.

2. *Cæsarean Section.* BELLUZZI. (*Gazz. Med. delle Calabrie*, Jan., 1876.)

A young woman, twenty-nine years old, pregnant and at term, entered the Maternity at Bologna. She was rachitic, and the conjugate diameter was estimated at about forty-eight millimetres. The fœtus was living, and presented by the vertex. The os had dilated to four centimetres, and the waters had escaped.

Under anæsthesia, the intestines were discovered (by palpation and percussion) over the right side of the uterus. These were pressed away, as far as possible, and the incision made somewhat to the left, parallel to the lenia alba. The feet of a living and active fœtus were seized, and the body withdrawn from the uterine cavity, as well as the entire placenta; but, in spite of the utmost care, the intestines escaped from the womb and were replaced with difficulty. The mother entered upon convalescence but soon died from metro-peritonitis. The child survived.

The pelvis of the dead woman examined, *post-mortem*, was found to measure in its conjugate diameter hardly 50 millimetres.

3. *Cæsarean Section, with Fortunate Issue both to Mother and Child.* TESTA. (*La Clinica, of Naples; Gazzetta Medica delle Calabrie*, Jan., 1876.)

A Neapolitan woman, thirty-two years old, had a pelvis whose lesser diameter measured scarcely ten lines. On the 2d of January, Prof. Cav. Raffælo Novi performed the Cæsarean section, in the presence of many professors and medical students, with the most brilliant results. From the time of etherization to the passing of the last suture, scarcely twenty minutes intervened. The incision of the uterus was made directly over the placenta. The fœtus was extracted living and active, and the mother was rapidly restored to health. This is the second successful operation recorded in Naples, and the happy results are thought due in part to the slight modification of the ordinary operation introduced by Prof. Novi.

4. *Deformity of Hand, resembling Foot, with Presentation in Labor.* COULON. (*Le Progrès Méd.*, No. 5.)

In a case of labor at term, with presentation of the trunk and fœtus long dead, version was attempted when dilatation was complete. When the hand entered the uterus, absence of the thumb was determined, with the presence of a limb bent at right angles, terminating in an osseous projection which seemed to be the calcaneum. C. attempted version with the limb, and failed. Ficheux was summoned, who encountered similar difficulty, and then, abandoning this limb, sought and found another, by means of which the labor was terminated.

The fœtus was found to be at term and of normal size. The right hand was normal in its direction, but not provided with a thumb. This appendage was also wanting in the left hand, which latter was also directed backward to the ulnar side, so that the ulnar border of the anterior part of the carpus and a portion of the metacarpus corresponded to the surface of the radio-ulnar articulation, the latter being represented merely by a small facet. The radius was almost entirely wanting. The ulnar border of the carpus presented also an articular facet. The superior face of the carpus was contiguous with the external border of the forearm, and projected beyond it, as the os calcis in the foot.

Hence the difficulty in recognizing the presentation.

5. *Treatment of Asphyxiated New-born Children by Allowing Blood to Escape from the Cord.* BUDIN. (*Le Progrès Méd.*, Jan. 15, 1876.)

The author clearly describes the distinction between blue asphyxia and white asphyxia of the newly-born. He also points out the danger to these infants from the induced hæmorrhage. They are left pallid and in a condition of "profound apathy."

When section of the cord is made at once, the infant is deprived of about 92 grammes of blood which should have been received from the placenta. If from two to four spoonsful more (40 to 80 grammes) be permitted to flow out, a new cause of profound anæmia comes into play. The infant then suffers a loss which in the adult would amount to 3,000 grammes. And all this for what? To relieve, you say, "pulmonary and cerebral congestion."

Pulmonary congestion can not evidently occur, since at birth there is atelectasis. As to cerebral congestion, that is only too readily mistaken for asphyxia. But even supposing there is congestion, let the infant be left attached to the cord and suffered to cry and respire freely. The cyanosis constantly disappears. The dilating lungs constitute a diverticulum into which the blood speedily pours; mixed with air in the pulmonary vesicles, this blood is rapidly oxygenated. The asphyxia and violet tint of the integument then disappear. If, on the other hand, bleeding from the cord is practiced, the hue of asphyxia disappears, it is true, but the skin, instead of assuming the delicate rose tint which is habitual, soon exhibits an extreme pallor.

The following conclusion is formulated: "In cases of asphyxia of the newly-born, before ligating and dividing the umbilical cord we should wait, if possible, for the establishment of respiration and the beating of the heart. *Bleeding from the cord should never be practiced before artificial respiration and insufflation have been tried*, in cases of apparent death.

6. *Best Period for Ligature of the Funis.* BUDIN. (*Gaz. Méd.*, Jan., 1876.)

At a recent meeting of the Société de Biologie, M. Budin read a communication (published in full in the *Progrès Médical* for January), giving an account of numerous investigations he had carried on in the Maternité, at the desire of M. Tarnier. From these it results that he is of opinion that the ligature and section of the cord should not be practiced until one or two minutes after the cessation of the pulsations in the cord, a very much larger portion of the circulating blood being retained in the system of the infant than when the cord is at once tied and divided. Moreover, the detachment and expulsion of the placenta, according to M. Tarnier's experience, seem to take place more readily when the infant has been allowed to breathe and cry for some time before its separation is effected.

7. *Congenital Absence of Uterus.*

C. C. Lewis, of Owensboro, Ky., reports a case of congenital absence of uterus, in a woman twenty-eight years of age, married three years. She, of course, has never menstruated, and has never experienced sexual desire. She has a vagina about three inches long, terminating in a cul-de-sac. She has well-developed breasts.

